# Potential therapeutic targets for ALS: *MIR206*, *MIR208b* and *MIR499* are modulated during disease progression in the skeletal muscle of patients

**DOI:** 10.1038/s41598-017-10161-z

**Published:** 2017-08-25

**Authors:** Lorena Di Pietro, Mirko Baranzini, Maria Grazia Berardinelli, Wanda Lattanzi, Mauro Monforte, Giorgio Tasca, Amelia Conte, Giandomenico Logroscino, Fabrizio Michetti, Enzo Ricci, Mario Sabatelli, Camilla Bernardini

**Affiliations:** 1 Institute of Anatomy and Cell Biology, Università Cattolica del Sacro Cuore, 00168 Rome, Italy; 2 Institute of Neurology, Università Cattolica del Sacro Cuore, Fondazione Policlinico Universitario “A. Gemelli”, 00168 Rome, Italy; 3 NEuroMuscularOmnicentre (NEMO), Fondazione Serena Onlus, 00168 Rome, Italy; 4 Institute of Orthopedic Clinic, Fondazione Policlinico Universitario “A. Gemelli”, 00168 Rome, Italy; 5 IRCCS San Raffaele Scientific Institute, Università Vita-Salute San Raffaele, 20100 Milan, Italy

## Abstract

Amyotrophic lateral sclerosis (ALS) is characterized by the progressive loss of motor neurons followed by muscle weakness, paralysis and death. The disease progression is extremely variable among patients, and reliable prognostic markers have not been identified. The aim of the study was to functionally characterize selected genes and microRNAs acting in the skeletal muscle of ALS patients, taking into account the duration and evolution of the disease, in order to obtain information regarding the muscle response to ALS progression. This prospective, longitudinal study enrolled 14 ALS patients and 24 age- and sex-matched healthy controls. Gene expression and histological analysis indicated an increase of *MIR208B* and *MIR499* levels and the predominance of slow fibres, respectively, in the muscles of patients with a slower disease progression. A decreased expression of *MIR206* and increased levels of HDAC4, during the progression of the disease were also observed. Taken together, our data suggest that the molecular signalling that regulates re-innervation and muscle regeneration is hampered during the progression of skeletal muscle impairment in ALS. This could provide precious hints towards defining prognostic protocols, and designing novel tailored therapeutic approaches, to improve ALS patients’ care and delay disease progression.

## Introduction

In amyotrophic lateral sclerosis (ALS) motor neuron degeneration leads to muscle atrophy, with death usually occurring as a result of respiratory failure, in 3–5 years. However, the survival time is extremely variable; some patients can live beyond 10 years since diagnosis, while others undergo a very rapid disease progression.

The first disease event, observed in both patients and transgenic mouse models, is the destruction of the neuromuscular junction. This led to formulate the “dying-back” hypothesis, according to which defects of neuromuscular functions occur before motor neurons (MNs) anomalies^1, 2^. Several pieces of evidence indeed indicate that, before the clinical onset and during the disease progression, the skeletal muscles of ALS patients undergo futile cycles of denervation and re-innervation, along with MNs degeneration^3^. When the MNs start degenerating, surrounding axons sprout from surviving neurons to re-innervate the muscle fibres and compensate the missing synapses. Muscle fibres are broadly classified into slow-twitch (type I) and fast-twitch (type II) based on the myosin heavy chain (*MHC*) gene expression and on the metabolic path (oxidative/glycolytic) used for ATP production^4^. Since all muscle fibres in a motor unit (MU) are of the same type, when the MUs enlarge, the skeletal muscle is reorganized in clustering of fibres of the same metabolic type (fibre type grouping). Ultimately these MUs lose their innervation and start undergoing atrophy. Type I and type II fibres are innervated by different MNs; interestingly both in ALS patients and in the SOD1G85R transgenic mouse model, the number of large-calibre axons (innervating type II fibres) in the spinal cord is significantly lower than in controls, whereas the number of small-calibre axons (innervating type I fibres) is preserved^5, 6^. It is noteworthy that specific muscles (e.g. the extrinsic eye muscles and the bladder detrusor muscle) are selectively spared in ALS, suggesting that the corresponding MNs, in charge of their innervation, are relatively resistant to neurodegeneration. Consistently with these observations, in the SOD1G93A mouse, muscles enriched in slow-twitch fibres undergo denervation at later stages, compared with those housing more fast-twitch fibres^7^. A strategy designed to target early muscle denervation could plausibly represent a valuable tool to delay disease progression.

Preclinical evidence obtained in the SOD1G93A mouse model indicated that the histone deacetylase 4 (HDAC4) increases in the skeletal muscle during the denervation process, and constitutively blocks the re-innervation signalling^8^. In turn, denervation induces the up-regulation of *MIR206*, which promotes re-innervation by suppressing muscular HDAC4 protein levels. Accordingly, in the SOD1G93A mouse model, suppression of the *MIR206*-dependent HDAC4 down-regulation accelerates disease progression, whereas *MIR206* over-expression boosts the regeneration of neuromuscular synapses after acute nerve injury^8^. HDAC4 inhibition through *MIR206* has been, indeed, proposed as a reasonable therapeutic strategy in ALS. In ALS patients, the muscular *HDAC4* levels positively correlate with the disease progression and severity, thus supporting its role also in human tissues^9^. Taken together, these observations suggest that the skeletal muscle, and the local molecular signalling, may represent valuable therapeutic targets in ALS^10^.

The aim of this study was hence to define the molecular signalling acting in the skeletal muscle of patients during the disease progression, in the attempt to identify suitable molecular markers implicated in skeletal muscle resistance to denervation and atrophy, and in the modulation of muscle re-innervation during early stages of ALS. To address these issues, we have focused on: 1. the *MIR206*/HDAC4 interplay underlying the human skeletal muscle response during ALS evolution; 2. the structural changes that occur during the disease progression in the muscle tissue of ALS patients, stratified per relevant clinical variables. The functional role of *MIR206* has been further validated *in vitro*, using primary cultures of satellite cells isolated from the skeletal muscle of ALS patients and controls.

## Results

### Clinical stratification of ALS patients

Based on the available information (see Table 1) about the clinical evolution of the disease, ALS patients were categorized into two groups: ‘*slow’*, i.e. ≥4 years of duration of disease without requiring respiratory supports (N = 6), and ‘*rapid’* (N = 5), i.e. <4 years of disease progression without respiratory support or death occurring <4 years from symptoms onset. The average disease duration significantly changed between the two groups (P = 0.0269), being 66.5 months (range 48–134) and 25 months (range 12–39), in the *slow* and *rapid* group, respectively. Patients in the *slow* group showed a significantly reduced age of onset (mean age = 50.5 years), compared with those in the *rapid* group (mean age = 65 years, P = 0.0466). ALS patients were then alternatively stratified based on the duration of the disease at biopsy (i.e. time passed since the onset of symptoms until the muscle biopsy). According to this criterion, patients were stratified into ‘*early’*, i.e. less than one year (N = 4) and ‘*late’*, i.e. more than one year (N = 9).

**Table 1:** Detailed ALS patient and specimen data.

Patient (Number)	Sex	Age at onset (years)	Age at biopsy (years)	Site of onset	Familiar recurrence	Time from onset to biopsy (months)	Patients stratification based on duration of disease at biopsy	Site of biopsy	MRC score*	ALSFRS-R**	Disease duration (months)	Patients stratification based on evolution of the disease	Gene mutation	Gene expression	MicroRNAs expression	Cell Cultures	Protein expression	Histology
1	F	50	NA	Spinal	Sporadic	NA	NA	Deltoid	F = 0	NA	134 (T)	*slow*	Negative	x	x			x
2	F	70	71	Spinal	Sporadic	13	*late*	Quadriceps	F = 5	33/48	30 (D)	*rapid*	Negative	x	x			x
3	M	69	71	Spinal	Sporadic	18	*late*	Deltoid	NA	NA	16 (T)	*rapid*	Negative	x	x			x
4	M	63	63	Spinal	Sporadic	7	*early*	Quadriceps	F = 5	36/48	28 (D)	*rapid*	Negative	x				x
5	M	65	65	Spinal	Sporadic	6	*early*	Deltoid	F = 3	42/48	59 (D)	*slow*	Negative	x	x			
6	M	43	43	Spinal	Sporadic	9	*early*	NA	NA	37/48	14 (LF)	NA	Negative	x	x			
7	F	59	59	Spinal	Sporadic	10	*early*	Quadriceps	F = 5	42/48	58 (D)	*slow*	*C9ORF72*	x	x			x
8	F	54	55	Bulbar	Sporadic	19	*late*	Deltoid	F = 5	43/48	39 (D)	*rapid*	Negative	x	x			x
9	M	50	53	Spinal	Familial	28	*late*	Quadriceps	F = 5	41/48	48 (A)	*slow*	Negative	x	x	x	x	x
10	F	28	30	Spinal	Sporadic	26	*late*	Quadriceps	F = 5	41/48	48 (A)	*slow*	Negative	x	x	x	x	
11	M	69	70	Bulbar	Sporadic	13	*late*	Quadriceps	F = 4	23/48	12 (T)	*rapid*	Negative	x	x	x	x	x
12	F	51	54	Spinal	Sporadic	36	*late*	Quadriceps	NA	NA	52 (A)	*slow*	Negative	x	x	x		
13	F	55	56	Spinal	Familial	14	*late*	Quadriceps	F = 3	43/48	25 (A)	NA	*C9ORF72*	x		x	x	
14	F	57	57	Spinal	Familial	13	*late*	Quadriceps	F = 5	46/48	18 (A)	NA	*SOD1 L84F*	x	x	x	x	

*The MRC score was referred to the muscle at biopsy and ranged from 0 (no visible movement) to 5 (normal muscle strength). **ALSFRS-R scores, at the time of muscle biopsy, ranged from 0 (total disability) to 48 (no disability). F = female; M = male; NA = not available; T = invasive mechanical ventilation by tracheotomy; D = dead; LF = lost at follow-up; A = alive.

### Gene and protein expression in skeletal muscle tissues

**Figure 1:**
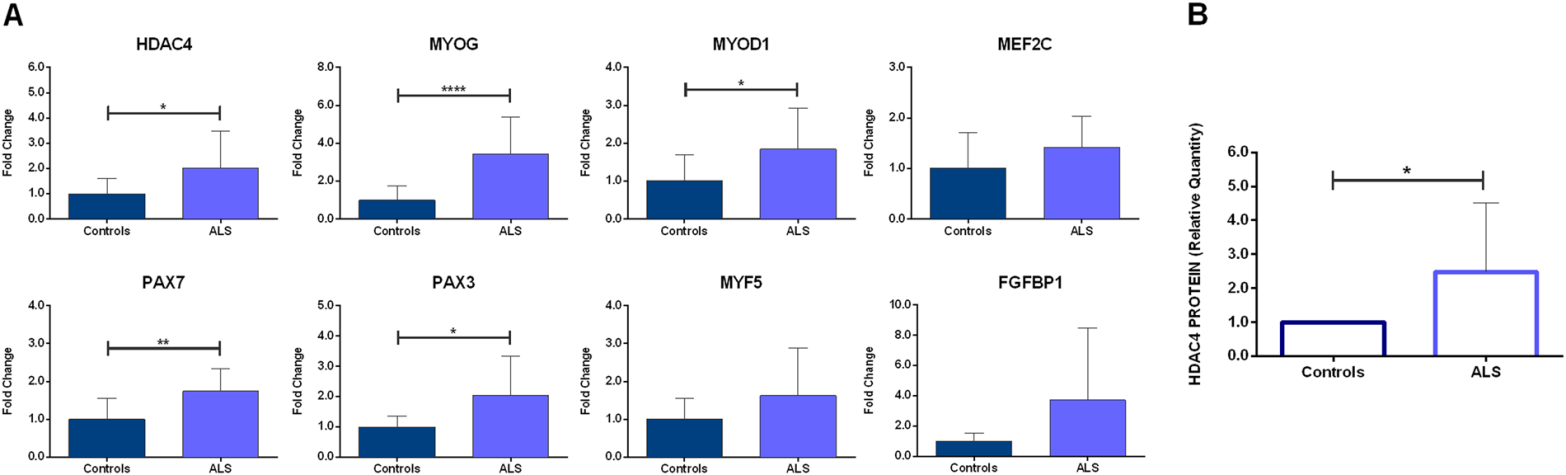
Expression profile of skeletal muscle biopsies of ALS patients and controls. (**A**) The diagrams plot the relative transcript levels of the genes analyzed by reverse qPCR, calculated according to the 2^−ΔΔCt^ method, using *ACTB* as housekeeping gene for normalization. All data were expressed as mean fold change ± SD across replicates, with control values set to 1. Unpaired t-test was used to detect the statistical significance between ALS and controls values; *P ≤ 0.05, **P ≤ 0.01, ****P ≤ 0.0001. (**B**) Quantitative analysis of western blot for HDAC4 protein expression in muscle specimens of ALS patients and controls. Relative protein content was determined by optical density of the HDAC4 bands normalized to stain free gel.

Eight genes and eleven microRNAs involved in the HDAC4 regulatory signalling have been analyzed in all the available tissue samples by qPCR. *HDAC4*, *MYOG*, *MYOD1*, *PAX7* and *PAX3* transcript levels resulted significantly increased in ALS patients compared with controls (Fig. 1A). Consistently, also HDAC4 protein levels increased in ALS samples compared with controls (Fig. 1B). None of the analyzed microRNAs showed a significant difference in expression levels, between patients and controls (Supplementary Fig. 1).

**Figure 2:**
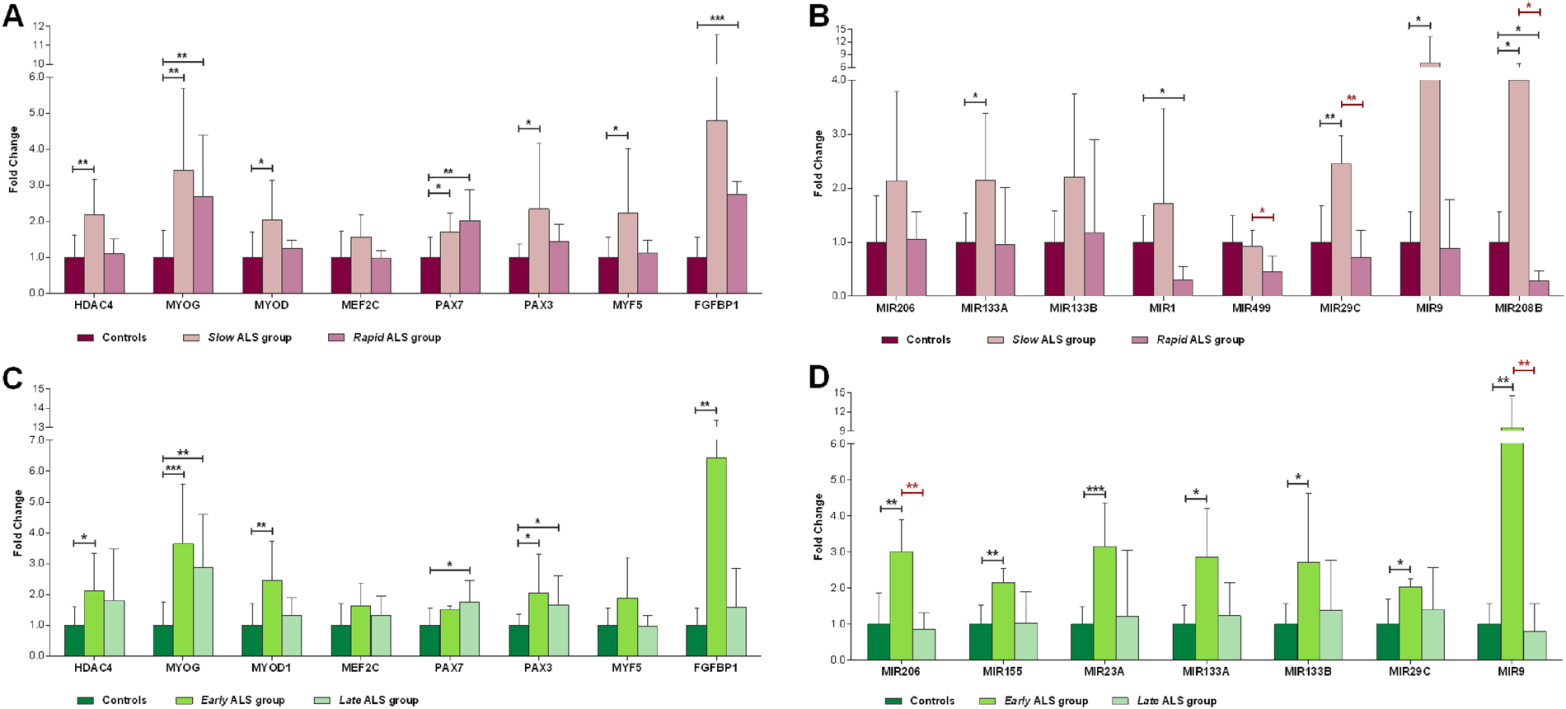
Molecular evaluation on the basis of the clinical stratification of ALS patients. Relative expression trend of genes (**A**) and microRNAs (**B**) in *slow* (with ≥4 years of disease progression without requiring respiratory support) and *rapid* (with <4 years of disease progression) ALS groups compared with controls. Relative expression trend of genes (**C**) and microRNAs (**D**) of *early* (less than one year from symptom onset to muscle biopsy) and *late* (more than one year from symptom onset to muscle biopsy) ALS groups and controls. All data were expressed as mean fold change ± SD across replicates, with control values set to 1. Unpaired t-test was used to detect the statistical significance between ALS and controls values; *P ≤ 0.05, **P ≤ 0.01, ***P ≤ 0.001.

Thereafter, gene expression data were also analyzed considering patients’ stratification. Results of differential gene expression according to the disease duration (i.e. patients categorized into *slow* and *rapid* groups) are displayed in Fig. 2A and B. In particular, *HDAC4*, *MYOD1*, *PAX3* and *MYF5* were up-regulated only in the *slow* group, compared with controls, while *MYOG* and *PAX7* increased in both groups. *FGFBP1* levels were very variable, reaching a significant up-regulation over controls only in the *rapid* group. Among the tested microRNAs, *MIR133A*, *MIR29C*, *MIR9* and *MIR208B* were significantly up-regulated in the ALS *slow* group, whereas *MIR1* and *MIR208B* expression was lower in the *rapid* group, than in controls. Interestingly, the expression of *MIR499*, *MIR29C* and *MIR208B* was significantly lower in the ALS *rapid* group than in the *slow* one.

**Figure 3:**
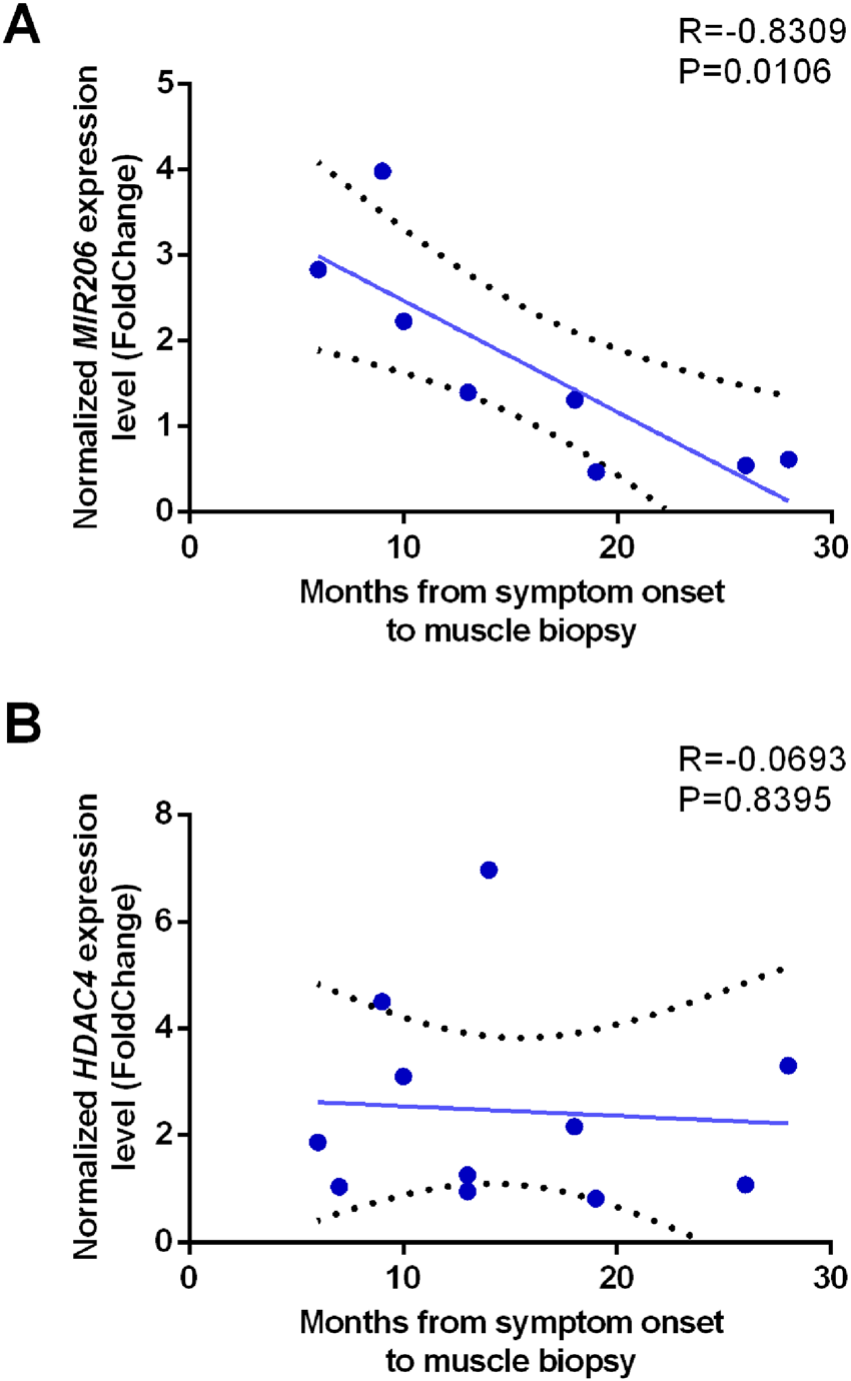
Correlation and expression analysis based on *MIR206* expression levels. Pearson’s correlation test of *MIR206* (**A**) and *HDAC4* (**B**) relative expression levels of ALS patients with the time from symptoms onset to muscle biopsy.

We then comparatively analyzed gene expression data in patients stratified based on disease duration at biopsy (i.e. within *early* and *late* patients’ groups). The results showed that, within one year from the onset of symptoms, the genes involved in regeneration (*PAX7* and *PAX3*) and re-innervation (*HDAC4*, *MYOG*, *MYOD1* and *FGFBP1*) pathways are activated in the affected skeletal muscle (Fig. 2C). The expression of *MIR206*, *MIR155*, *MIR23A*, *MIR133A*, *MIR133B*, *MIR29C* and *MIR9* increased only in the *early* stage group, compared with controls (Fig. 2D). *MIR206* and *MIR9* expression levels also significantly changed between the *early* and the *late* groups. Interestingly, *MIR206* levels inversely correlated with the time from symptoms onset to muscle biopsy (R = −0.8309, P = 0.0106) (Fig. 3A). Conversely, *MIR9* expression did not correlate with the progression of the disease (data not shown). Also, we did not find any statistically significant correlation between either of HDAC4 transcript (Fig. 3B) and protein levels and the disease duration (data not shown).

### Gene expression during *in vitro* myogenic differentiation

**Figure 4:**
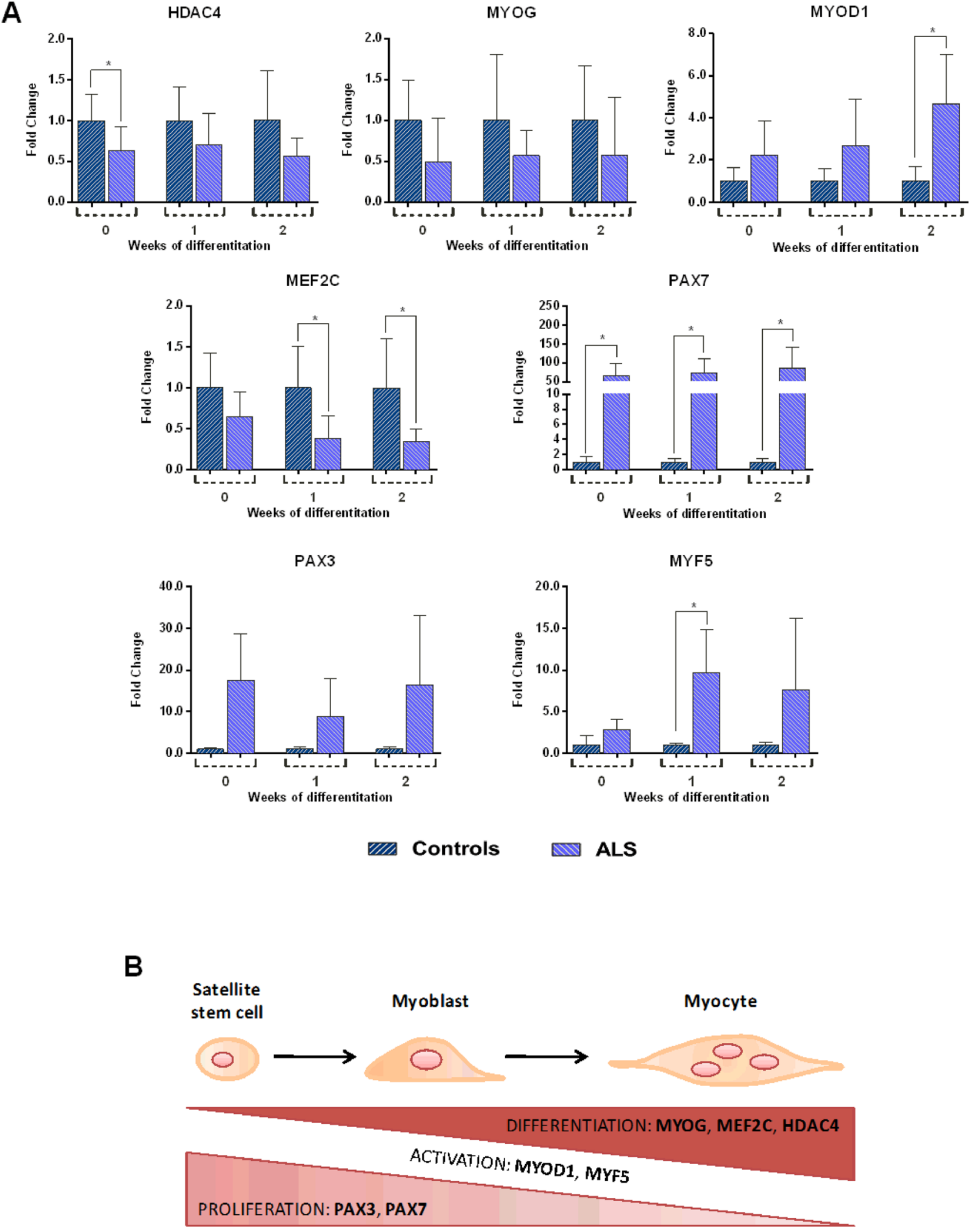
ALS and controls cell cultures display a different response to myogenic induction. (**A**) Relative transcript levels of the genes involved in proliferation, activation and differentiation processes, analyzed in time course. All data were expressed as mean fold change ± SD across replicates, with control values set to 1. Unpaired t-test was used to detect the statistical significance; *P ≤ 0.05. (**B**) Scheme of the putative molecular signature of myogenic conversion: the expression of differentiation markers increase while satellite cells turn into terminally differentiated myocytes, at the start of the myogenic induction and after 1 and 2 weeks.

Satellite cells derived from ALS patients and controls were induced towards myogenic differentiation *in vitro* for 14 days. Thereafter, the expression of the genes involved in the myogenic differentiation process (*HDAC4*, *MYOG*, *MYOD1*, *MEF2C*, *MYF5*, *PAX7* and *PAX3*) was analyzed in time course, after 1 and 2 weeks of induction. The expression trends, during *in vitro* differentiation, were very similar in ALS and controls for all tested genes (Supplementary Fig. 2). When comparing the gene expression levels between cells from ALS patients and controls at each time point (i.e. expression analysis between matched ALS and control cultures at each single time point), we found significant differences in selected genes: *MYOD1*, *PAX7*, *PAX3* and *MYF5* showed increased levels in ALS patients, compared with controls, while *HDAC4*, *MYOG* and *MEF2C* levels were lower in the ALS group than in controls (Fig. 4A). *PAX7*, *MYOD1* and *MYF5* represent early markers of stem cell activation, while *MYOG*, *MEF2C* and *HDAC4* are known to increase in later stages of the differentiation process (Fig. 4B). Therefore, the expression profiles observed in our sample seemed to suggest that the myogenic differentiation slows down in ALS cells compared with controls.

### Functional characterization of *MIR206* in cells

**Figure 5:**
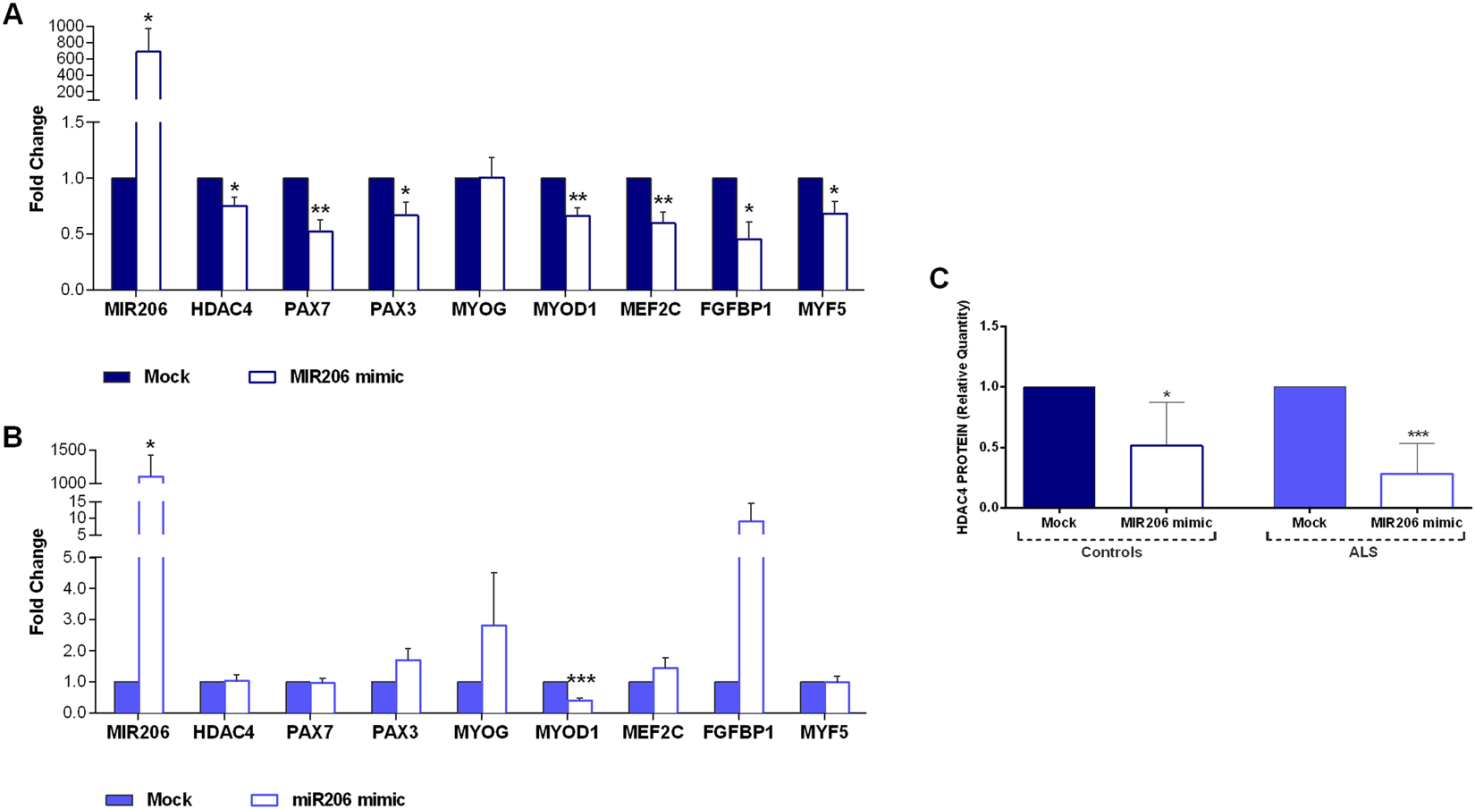
*MIR206* mimic effects in primary cultures of ALS patients and controls. Controls and ALS cultures induced along myogenic differentiation, were transfected with *MIR206* mimic for 1 week. The graphs show the effects of *MIR206* over-expression on the expression of the genes involved in regeneration and innervation pathways, in controls (**A**) and ALS (**B**) cultures. All data are expressed as mean fold change ± SD across replicates, with mock treated cells values set to 1. Unpaired t-test was used to detect the statistical significance between samples; *P ≤ 0.05, **P ≤ 0.01, ***P ≤ 0.001. (**C**) Quantitative analysis of western blot for HDAC4 protein expression in ALS patients and controls cultures treated upon *MIR206* over-expression. HDAC4 relative protein content was determined by optical density of the HDAC4 bands normalized to stain free gel.

In order to validate the functional effect of *MIR206* in the myogenic process, we performed a *MIR206* mimic assay in ALS and control satellite cells. The mimic construct was transfected in cells during the administration of myogenic medium for 1 week. The expression of the tested targets (*HDAC4*, *PAX7* and *PAX3* genes) and of the genes involved in the *MIR206*/*HDAC4* interplay (*MYOD1*, *MEF2C*, *MYF5* and *FGFBP1*) was significantly reduced upon the *MIR206* mimic treatment in control cells (Fig. 5A). In ALS cell cultures, only *MYOD1* levels significantly decreased after *MIR206* mimic, while *MYOG* and *FGFBP1* showed increased expression trends (Fig. 5B). HDAC4 protein levels were significantly reduced after *MIR206* mimic treatment, in both ALS and controls (Fig. 5C). Therefore, although *HDAC4* transcript levels did not change, *MIR206* over-expression was apparently able to modulate protein expression also in ALS cells, during the course of myogenic differentiation.

### Morphological assessment of skeletal muscles

**Figure 6:**
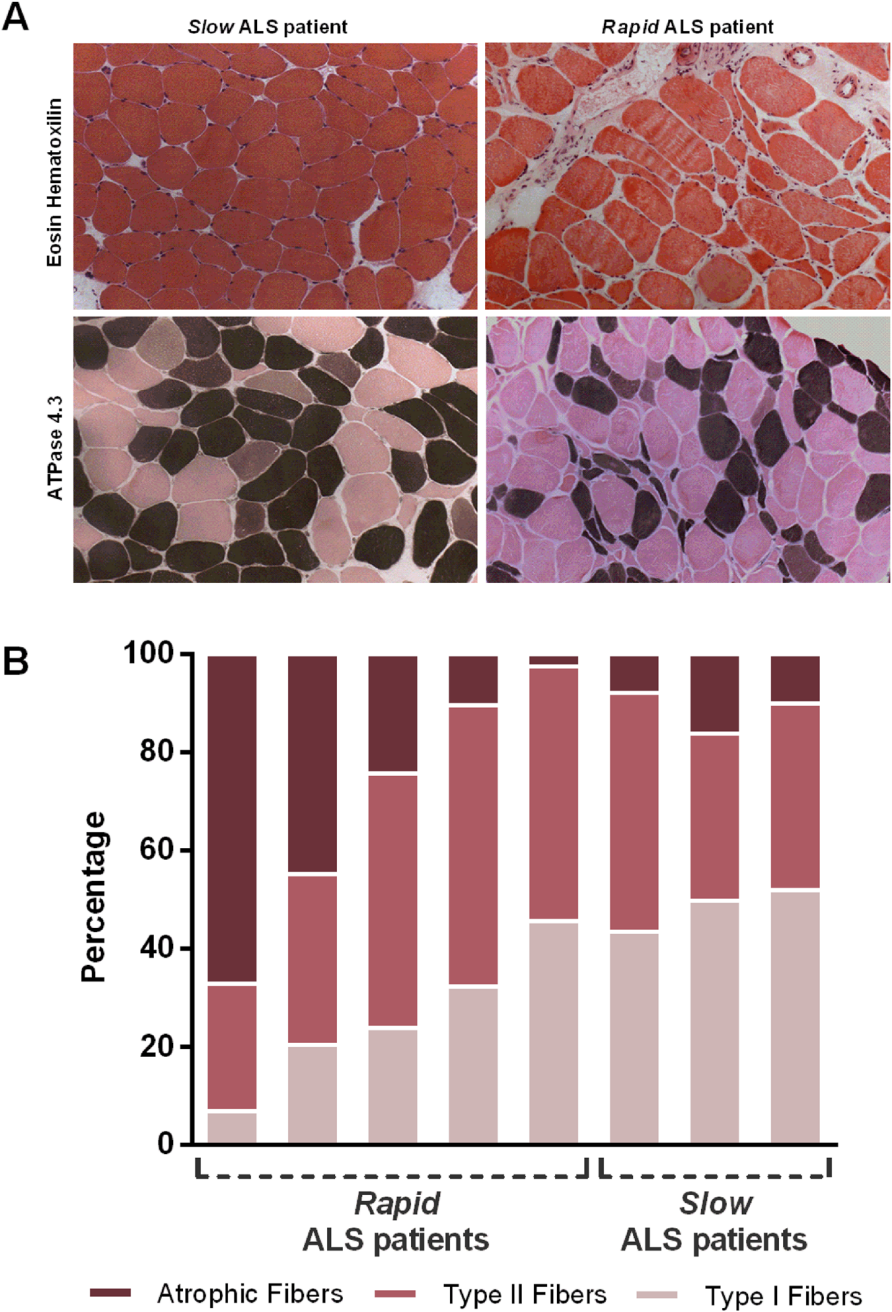
Histological analysis of ALS patients. (**A**) Representative images that showed the pattern of muscle fibre atrophy, with different fibre types stained differently. Differences in the fibre type ratio is detected between ALS patients in the *slow* group (left panels) and those in the *rapid* group (right panels). The hematoxylin-eosin staining (top panels) and the ATPase staining (bottom panels) highlighted the atrophy pattern and fibre composition. (**B**) The bar-graph illustrates the percentage of type I, type II and atrophic fibres in the skeletal muscle sections of *rapid* and *slow* ALS patients. An overall number of >1000 muscle fibres was counted for each assessed patient.

The down-regulation of *MIR208B* and *MIR499* observed in the *rapid*-versus-*slow* progression groups, is known to be associated with fibre type switch in the skeletal muscles^11^. This prompted us to perform the morphological assessment comparing patients in the *rapid* group (N = 5) with those in the *slow* group (N = 3). To define the distribution of fibre types I and II, and the percentage of atrophic fibres, we performed histological analysis on skeletal muscle biopsies from quadriceps and deltoid muscles of ALS patients (Fig. 6A).

The results of fibre quantification (Fig. 6B and Supplementary Table 1) showed that the percentage of type I fibres is greater in the *slow* than in the *rapid* group (mean 48,38–25,84% respectively, P = 0.0421). The percentage of type II fibres did not change between the groups. Interestingly, in the *rapid* group the percentage of atrophic fibres inversely correlated with the amount of both types of fibres (R_I fibres_ = −0.9548, P = 0.0115; R_II fibres_ = −0.947, P = 0.0145), whereas in the *slow* group, it inversely correlated only with the amount of type II fibres (R_II fibres_ = −0,864, though this correlation is not significant). Our data indicated also that the percentage of type II fibres in the *rapid* group is lower than the percentage of type I fibres, the latter being mainly spared from atrophy in the *slow* group, while preferentially damaged in the *rapid* one.

## Discussion

Amyotrophic lateral sclerosis (ALS) is a fatal neurodegenerative disorder, for which, to date, no effective therapy has been found. The mean survival of patients from symptom onset is 3 to 5 years^12^, but the disease progression and severity are extremely variable. Some patients have a milder and protracted course of illness, while others need respiratory support as early as within one year from the onset. An exact estimate of the rate of long surviving patients is not available; recent population-based studies have reported a 5-years survival rate ranging from 22 to 52% of patients^13^. Several variables, including predominance of upper motor neuron signs, spinal onset, male sex and younger age, have been already analyzed to establish associations with a longer life expectancy, but the results are not univocal. Based on the extant literature, the age and site of symptoms’ onset are the strongest prognostic predictors. In fact, in several population-based studies, younger age at onset has been found to predict long-term survival of ALS patients, whereas bulbar onset has been invariably identified as a negative prognostic predictor^13^.

In this study, we have used an *in vitro* model to investigate the response of the skeletal muscle during the disease progression. We have specifically found that the skeletal muscle of ALS patients showing a longer survival period expresses higher levels of microRNAs involved in the regulation of the slow-to-fast fibre type switch profile, compared with patients with a rapid disease progression. Moreover, we observed that the expression of molecular markers of skeletal muscle denervation tends to decrease within the first year since clinical onset.

In particular, we demonstrated that genes encoding myogenic regulatory factors (*HDAC4*, *MYOG*, *MYOD*, *PAX3* and *PAX7*) are consistently up-regulated in the skeletal muscle of ALS patients, more intensely within the first year since the onset of symptoms. These data may suggest that during the early stages of ALS, the molecular signalling implied in muscular regeneration is activated. In addition, the expression of *MIR206* and *MIR9* was significantly different between patients in early and late stages of disease progression. These data further indicated that the skeletal muscle of ALS patients activates an early response to denervation, which decreases over time at later stages. Also *HDAC4* activation seemed to occur within one year from symptoms’ onset. *HDAC4* up-regulation has been already described as a response to skeletal muscle denervation in the SOD1G93A ALS mouse model, in which it induces *MYOG* up-regulation. This, on turn, triggers the molecular cascade associated with muscular atrophy^8^. *MYOG* activates, indeed, the transcription of *MIR206*, establishing a negative feedback loop able to inhibit *HDAC4*. Finally, *HDAC4* inhibition induces the expression of *FGFBP1*, which promotes re-innervation and regeneration of the neuromuscular junction^8^. In humans *HDAC4* is up-regulated in ALS patients featuring a rapidly progressive disease compared with long-term survivors, while *MIR206* is up-regulated in both patient groups, although its levels do not correlate with the disease progression^9^. Interestingly, our data indicated that *HDAC4* transcript and protein are increased in all patients, regardless of the time passed since the onset of symptoms at the time of muscle biopsy. Apparently, *HDAC4* levels grow up until six months of disease duration and then it reaches a constant trend. In the early stage of the disease, also *FGFBP1* and *MYOG* are activated demonstrating that the pathway described in mice^8^ is active also in humans. However, after one year from symptoms onset, *FGFBP1* and *MIR206* levels decrease, which may cause the muscle to be less prone to react to denervation and muscular atrophy. Indeed, our data indicated that *MIR206* up-regulation is detectable in the affected muscle within one year from the clinical onset, and becomes less evident as the disease progresses to a later stage.

In our study, the intracellular levels of *MIR206* could be efficiently modulated in ALS muscle satellite cells, in which *MIR206* over-expression is associated with HDAC4 protein down-regulation. Indeed, upon mimicking *MIR206* in cells, the expression of transcription factors downstream to *HDAC4* decreased.

Our data also showed an up-regulation of *MIR208B* and *MIR499* in the skeletal muscles of patients with a slow progression of the disease. *MIR208B* and *MIR499* play redundant roles in the specification of muscle fibre identity, by activating slow-, and repressing fast-myofibre molecular programs^11^. *MIR208B* and *MIR499* genes are encoded within introns of two type I Myosin Heavy Chain (MyHC) genes (*MYH7* gene coding for MyHC-β/slow and *MYH7B* gene coding for MyHC-slow tonic, respectively). Double knockout mice for both miRNAs displayed a marked slow-to-fast switch in fibre type profile and MyHC expression, while muscle-specific over-expression of *MIR499* led to a complete fast-to-slow switch in the MyHC profile^11^. On this regard, the histological analysis of muscle biopsies of ALS patients showed that type I fibres in the *slow* group are mainly spared from atrophy, while being preferentially damaged in the *rapid* one. Slow and fast fibres in the skeletal muscle are innervated by different MNs. Interestingly, both in ALS patients and in the SOD1G85R transgenic mouse model, the axons of neurons innervating type I fibres are selectively spared^5, 6^. Moreover, a recent study on the SOD1G93A mouse, demonstrated the occurrence of a fast-to-slow shift in the fibre type composition, at later stages of the disease^14^. Altogether these data suggested that the muscle fibres and the corresponding MNs collaborate to counteract skeletal muscle atrophy during early stages of ALS pathogenesis. This may lead to a slow muscle phenotype in *long* surviving patients, similarly to what occurs in mutant mice at final stages. The intrinsic properties of muscle cells, together with the pattern of impulse activity imposed on muscle fibres, play a dominant role in determining fibre type composition in regenerating muscles, which, in turn, respond differently to the same stimulation pattern^15^.

The skeletal muscle can regenerate after injury, due to the activation and proliferation of satellite cells. Normally quiescent, these myogenic precursors are activated during damage and start expressing specific myogenic transcription factors^16^. In line with previous evidence in the literature^17, 18^, our data indicated that satellite cells from ALS patients tend to respond slower to *in vitro* myogenic induction. These cells displayed opposite expression trends between genes associated with regeneration and early differentiation (*PAX3* and *PAX7*), and markers of terminal differentiation (*MYOG* and *MEF2C*). This may suggest that, as a result, the skeletal muscle could not be able to effectively repair and regenerate, leading to severe muscle atrophy and weakness. The muscle regeneration process is indeed hampered or, at least, delayed in ALS patients. Different studies performed on ALS satellite cultures, already demonstrated that ALS-derived myoblasts are unable to fully differentiate into myotubes^17, 18^, and that myotubes from ALS patients have decreased levels of the MYOG protein^18^.

In this scenario, *MIR206* seems to play an essential role during the pathogenesis of ALS skeletal muscle impairment and regeneration, by regulating *PAX7*, *PAX3* and *HDAC4* levels, hence altering the balance between cell proliferation and differentiation^19^.

**Figure 7:**
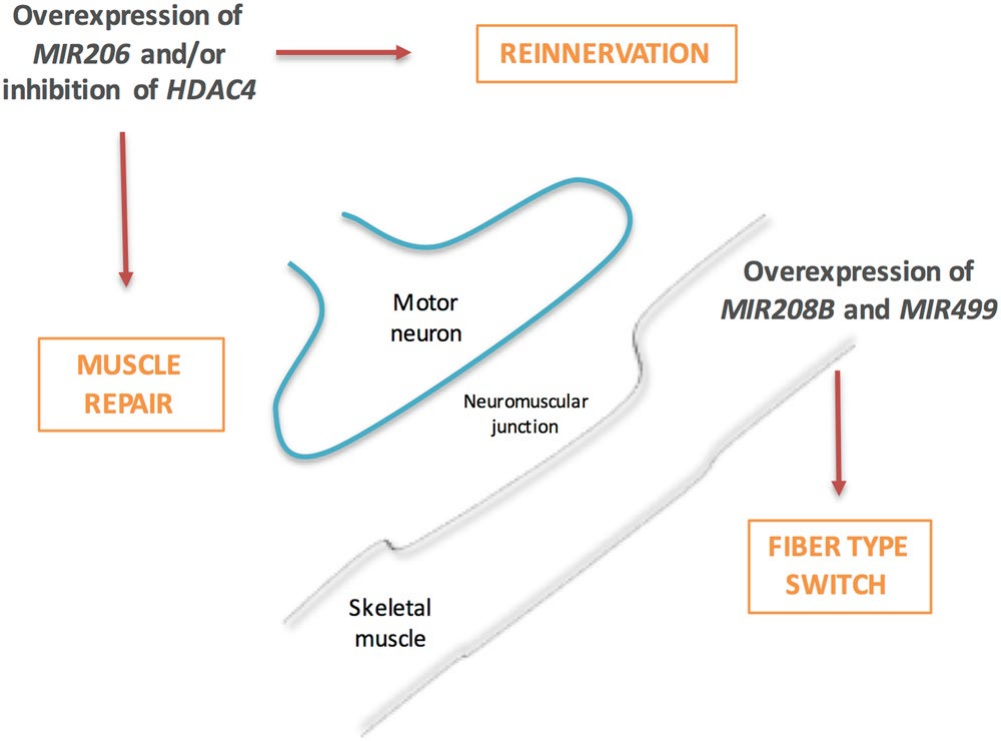
Schematic representation of possible therapeutic targets in the skeletal muscle at the neuromuscular junction. We identified three muscular microRNAs as potential therapeutic targets in ALS patients. Their targeted over-expression in the skeletal muscle could act on several fronts: (1) promote the regeneration process of the muscle itself, (2) increase the signals aimed at promoting the re-innervation by the motor neuron and (3) lead to a slowdown of the atrophy process by promoting the conversion of the muscle fibres in the more resistant slow type.

In conclusion, our study allowed identifying a complex molecular network acting in the skeletal muscle of ALS patients, plausibly affecting both re-innervation and muscle regeneration. Re-innervation and muscle repair are inherently inter-dependent processes, as the skeletal muscle plays an active role in maintaining neuromuscular junction stability. *MIR206* seems to link these processes, as its over-expression enhances the muscle regenerative potential during ALS progression^8^. An over-expression of *MIR208B* and *MIR499* could instead make the skeletal muscle more resistant to denervation and ALS progression (Fig. 7).

Targeting muscular microRNAs could represent a valuable strategy aimed at restoring the molecular pathways improving motor performance and enhancing the production of growth factors (such as *FGFBP1*) able to facilitate the re-innervation process and delay ALS progression.

## Materials and Methods

### Patients and specimens

14 ALS patients and 24 age- and sex-matched healthy controls have been enrolled in this study, which was approved by the ethics committee of the Università Cattolica del Sacro Cuore [protocol P/774/CE/2012]. All the following experiments were performed in accordance with relevant guidelines and regulations. All patients provided a written informed consent prior to all investigations. ALS diagnosis was performed according to the revised El Escorial criteria. The functional impairment was assessed using the revised ALS Functional Rating Scale (ALSFRS-R), which rates the performance of activities of daily living and scores from 0 (total disability) to 48 (no disability), based on a 12-item scale. Muscle strength was measured by manual muscle testing according to the grading system proposed by the Medical Research Council (MRC score), ranging from 0 (no visible movement) to 5 (normal muscle strength).

Detailed clinical data of ALS patients are provided in Table 1. Briefly, the ALS sample included six males and eight females, aged 30-to-71 (average age: 57 ± 12) years at biopsy. All patients were Caucasian. Genetic testing was performed to assess the presence of mutations in the ALS-associated *SOD1*, *TARDBP*, *FUS*, *ANG*, *ATXN2* and *C9orf72* genes. Three patients carried *SOD1* and C9orf72 mutations.

Age- and sex- matched control individuals were selected among patients undergoing orthopaedic surgery for traumatic injury and without positive history for muscle weakness, nor any neurological disorders.

A skeletal muscle specimen was collected through an open biopsy performed either in the deltoid muscle (four ALS patients) or in the quadriceps (nine ALS patients). Upon collection, the tissue specimens were stored in liquid nitrogen for histological analyses, in Allprotect tissue reagent (Qiagen) for protein isolation, in RNA later (Qiagen) for RNA extraction, and in culture medium for cells isolation.

### Cell cultures

Primary satellite cells were isolated from muscle specimens (six patients and six controls), through enzymatic digestion. Briefly the tissue was mechanically fragmented and digested with collagenase/dispase (Roche). The tissue homogenate was then separated through a 40 µm mesh filter and seeded into culture plates. Satellite cells were maintained in RPMI 1640 medium (EuroClone), supplemented with 1% L-glutamine (EuroClone), 1% antibiotics (penicillin 100 IU/ml, streptomycin 100 mg/ml, EuroClone) and 15% fetal bovine serum (Gibco).

The myogenicity of the cell populations was assessed by immunofluorescence using an antibody against desmin (Thermo Fisher Scientific, #PA5-17182), specifically expressed in myogenic cells, and counting the number of desmin-positive cells. Cultures displaying over 70% myogenic cells were selected for further *in vitro* experiments. To induce the myogenic differentiation, cells were shifted to RPMI 1640 medium with 5% horse serum (EuroClone).

### RNA isolation and quantitative Real Time PCR

Total RNA was isolated from muscle specimens and cultured cells using the TRIzol reagent method (Invitrogen), according to manufacturers’ protocols. RNA purity and concentration were quantified using an UV spectrophotometer (DU 800, Beckman Coulter). The expression of selected mRNA and microRNA was analyzed using reverse transcription quantitative real-timePCR (qPCR). 1 μg of total RNA was reverse-transcribed, using SuperScript III First-Strand Kit (Invitrogen), and subsequently quantified with StepOne system (Applied Byosistem), using specific FAM-labelled TaqMan probes (Thermo Fisher Scientific) for *ACTB*, *HDAC4*, *MYOG*, *MYOD1*, *MEF2C* and *PAX7*. For the determination of *MYF5*, *FGFBP1* and *PAX3* transcript levels, we used SybrGreen technology (Life Technologies).

For microRNAs analysis, 10 ng of total RNA was reverse-transcribed, using the TaqMan MicroRNA Reverse Transcription kit (Life Technologies), and subsequently quantified using specific FAM-labelled TaqMan probes (Thermo Fisher Scientific) for *U6*, *MIR206*, *MIR155*, *MIR23A*, *MIR133A*, *MIR133B*, *MIR1*, *MIR499*, *MIR208B*, *MIR29C*, *MIR9* and *MIR223*.

The FAM-labelled TaqMan probes and the sequences of the primers used in SybrGreen technology are listed in Supplementary Table 2. The 2^−ΔΔCt^ method was applied to calculate fold differences in gene expression using the housekeeping genes (*ACTB* and *U6*, for genes and microRNAs expression, respectively) for data normalization.

### Protein extraction and Western Blot analysis

20–30 mg of frozen tissues were homogenated in 200 μl modified lysis buffer (25 mM Tris-HCl pH 7.6, 2 mM EDTA pH 8, 250 mM Sucrose, 0.1% TritonX-100, 5 mM DTT, 1 mM PMSF, 200 mM SOV4, 10 mM NaF, protease inhibitor cocktail 1X; Sigma-Aldrich). A standard RIPA buffer (25 mM Tris-HCl, 1% NP-40, 1% Na-deoxycholate, 150 mM NaCl, 1 mM PMSF, 1 mM NaF, 1 mM Na3VO4, protease inhibitor cocktail 1X; Sigma-Aldrich) was instead used for the extraction of total proteins from cell cultures. Protein samples were quantified by the Pierce BCA Protein Assay Kit (Thermo Fisher Scientific) and stored at −20 °C.

20 µg of lysate samples were separated on Mini-PROTEAN TGX Stain-Free Gels (4–20%, Bio-Rad). The gel was transferred to a nitrocellulose membrane (Bio-Rad) for 10 min using the Trans-Blot Turbo Transfer System (Bio-Rad) with Trans-Blot Turbo Mini Transfer Packs (Bio-Rad). The membrane was activated for 1 minute and then incubated with an antibody against HDAC4 (Cell Signaling, #2072) at a dilution of 1:500. Immune complexes were detected using the ChemiDoc Touch Imaging System (Bio-Rad)after incubation with Clarity Western ECL Substrate (Bio-Rad). HDAC4 protein expression was quantified and normalized to stain free gel loading using ImageLab software (version 5.2.1, Bio-Rad).

### Histological analysis

Frozen biopsies were cut into 10 micrometers-thick slices using a cryostat. Sections were stained using the following histochemical techniques: hematoxylin and eosin, nicotinamide adenine dinucleotide dehydrogenase tetrazolium reductase and ATPase at pH 4.3, 4.6 and 9.4.An overall number of >1000 muscle fibres was counted for each assessed biopsy, using the Stereo Investigator system (Stereo Investigator software, Version 9). Fibres below 40 micrometers in diameter were considered atrophic.

### microRNA mimic treatments

MicroRNA mimicry was performed in primary satellite cell cultures. To this aim, cells were plated at a 50% seeding density, andgrownuntil 80–90% confluent. Myogenic induction was then initiated by replacing the growth medium with the differentiation medium (see above) for 1 week. The *MIR206* mimic (Qiagen), was added to medium [50 nM] and transfected in cells using the FuGENE HD transfection reagent (Promega), at the beginning and after 4 days of myogenic induction. Cells treated only with transfection reagent (mock) were used as controls. After 1 week, cells were collected and protein and RNA extracted; the effects of treatments were then evaluated by qPCR and western blot.

### Statistical analysis

Data were analyzed using GraphPad Prism software version 6.0. All data are shown as means ± standard deviation (SD). Statistical differences between groups were analyzed using unpaired Student’s t-test. Correlations were calculated using the Pearson’s correlation test. All statistics were two-tailed and the level of significance was set at P = 0.05.

## Electronic supplementary material

Supplementary Information
