# Supplementary material for: Potential therapeutic targets for ALS: MIR206, MIR208b and MIR499 are modulated during disease progression in the skeletal muscle of patients
Source: Sci Rep. 2017 Aug 25;7:9538. doi: 10.1038/s41598-017-10161-z (PMC5573384; doi:10.1038/s41598-017-10161-z)
Supplement: Supplementary file 1 — Supplementary Information [file 41598_2017_10161_MOESM1_ESM.pdf]

Potential therapeutic targets for ALS: *MIR206*, *MIR208b* and *MIR499* are modulated during disease progression in the skeletal muscle of patients

Lorena Di Pietro[1], Mirko Baranzini[1], Maria Grazia Berardinelli[1], Wanda Lattanzi[1], Mauro Monforte[2], Giorgio Tasca[2], Amelia Conte[3], Giandomenico Logroscino[4], Fabrizio Michetti[1,5], Enzo Ricci[2], Mario Sabatelli[2,3] and Camilla Bernardini[1]

[1]Institute of Anatomy and Cell Biology, Università Cattolica del Sacro Cuore, 00168, Rome, Italy

[2]Institute of Neurology, Università Cattolica del Sacro Cuore, Fondazione Policlinico Universitario "A. Gemelli", 00168, Rome, Italy

[3]NEuroMuscularOmnicentre (NEMO), Fondazione Serena Onlus, 00168, Rome, Italy

[4]Institute of Orthopedic Clinic, Fondazione Policlinico Universitario "A. Gemelli", 00168, Rome, Italy

[5]IRCCS San Raffaele Scientific Institute, Università Vita-Salute San Raffaele, 20100, Milan, Italy

Corresponding author

Dr. Camilla Bernardini,

Institute of Anatomy and Cell Biology, Università Cattolica del Sacro Cuore,

Largo Francesco Vito 1, 00168, Rome, Italy;

e-mail: camilla.bernardini@unicatt.it

Supplementary information

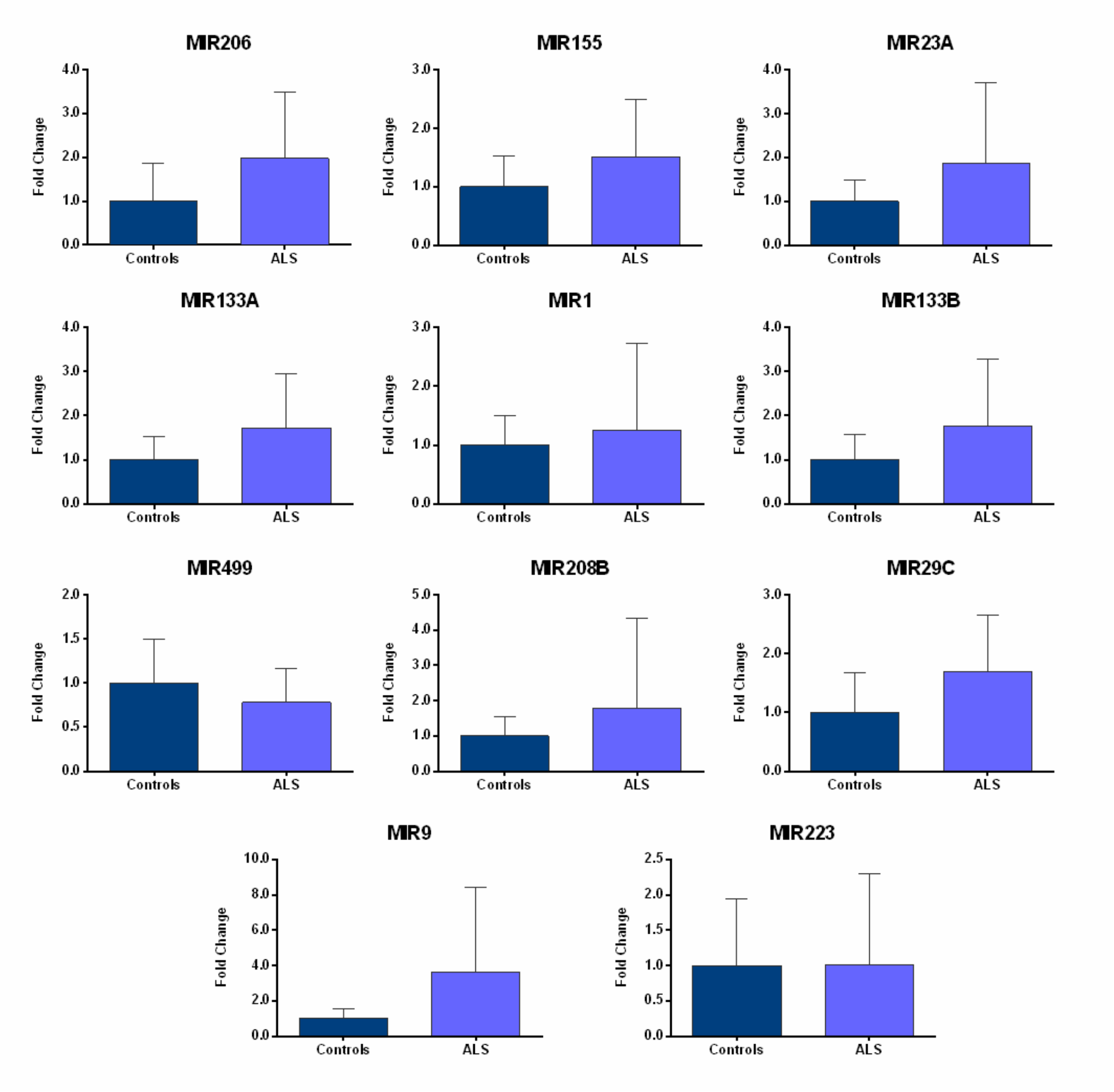

Supplementary Figure 1 Expression profile of selected microRNAs of skeletal muscle biopsies of ALS patients and controls. Relative transcript levels of microRNAs analyzed by reverse qPCR. *U6* is used for normalization and all data are expressed as mean fold change ± SD across replicates, with control values set to 1.

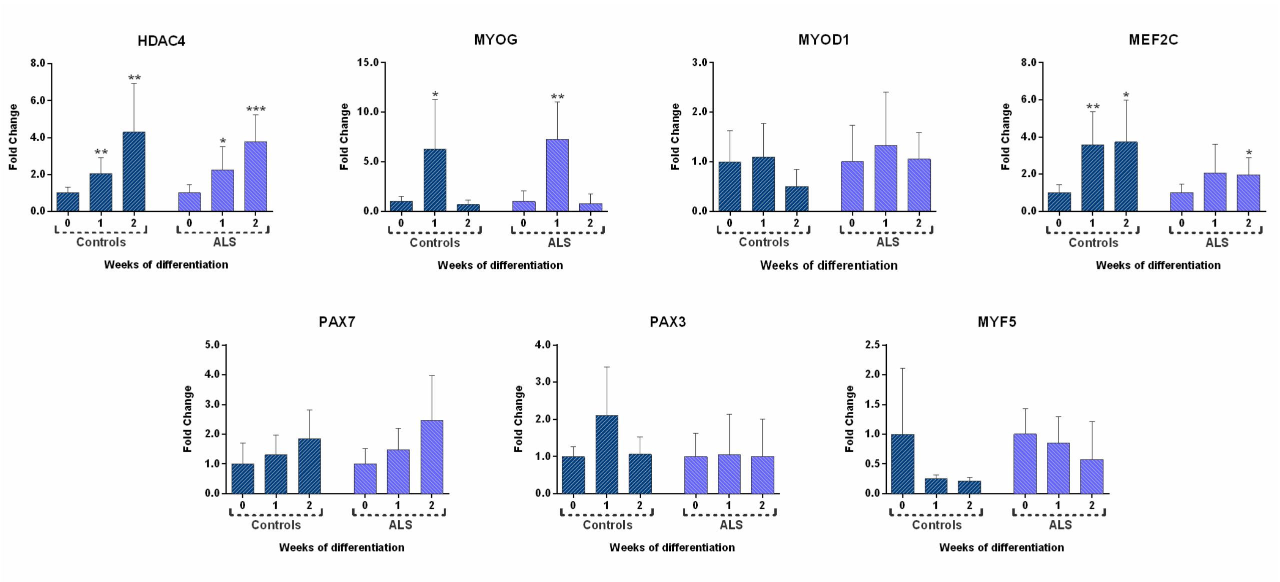

Supplementary Figure 2 ALS and controls cell cultures expression response to *in vitro* myogenic induction. Relative transcript levels of genes involved in myogenic differentiation carried out independently in controls and ALS patients cell lines. The graphs illustrate the relative quantity of gene levels after 1 and 2 weeks of differentiation, compared with the levels found at the beginning of the *in vitro* induction. *ACTB* is used for normalization and all data are expressed as mean fold change ± SD across replicates, with control values set to 1. Unpaired t-test was used to detect the statistical significance between ALS and controls values; *P≤0.05, **P≤0.01, ***P≤0.001.

Patient	Disease Progression	Type I Fibers	Type II Fibers	Atrophic Fibers
**3**	*Rapid*	6,92%	25,95%	67,13%
**11**	*Rapid*	20,40%	34,70%	44,90%
**4**	*Rapid*	23,96%	51,76%	24,28%
**2**	*Rapid*	32,31%	57,14%	10,54%
**8**	*Rapid*	45,60%	51,78%	2,61%
**7**	*Slow*	43,38%	48,67%	7,95%
**1**	*Slow*	49,84%	33,97%	16,19%
**9**	*Slow*	51,92%	38,01%	10,07%

Supplementary Table 1 Percentage of type I, type II and atrophic fibres in skeletal muscle sections of *slow* and *rapid* ALS patients.

Gene	FAM-labeled TaqMan probes
ACTB	Hs01060665_g1
HDAC4	Hs01041638_m1
MYOG	Hs01072232_m1
MYOD1	Hs02330075_g1
MEF2C	Hs00231149_m1
PAX7	Hs00242962_m1
RNU6B	Assay ID=001093
hsa-*MIR206*	Assay ID=000510
hsa-*MIR155*	Assay ID=002623
hsa-*MIR23A*	Assay ID=000399
hsa-*MIR133A*	Assay ID=002246
hsa-*MIR133B*	Assay ID=002247
hsa-*MIR1*	Assay ID=002222
hsa-*MIR499*	Assay ID=001045
hsa-*MIR208B*	Assay ID=002290
hsa-*MIR29C*	Assay ID=000587
hsa-*MIR9*	Assay ID=000583
hsa-*MIR223*	Assay ID=002295
**Gene**	**SybrGreen primers sequence**
*ACTB*_forward	TCGTGCGTGACATTAAGGAG
*ACTB*_reverse	CCATCTCTTGCTCGAAGTCC
*MYF5*_forward	ATGCCATCCGCTACATCGAG
*MYF5*_reverse	ATTCGGGCATGCCATCAGAG
*FGFBP1*_forward	TGCTCAGAACAAGGTGAACGC
*FGFBP1*_reverse	ACCACTTTGCTGTGAAGTCCA
*PAX3*_forward	CTGGAAGTGTCCACTCCCCTC
*PAX3*_reverse	CACGATCTTGTGGCGGATGT

Supplementary Table 2 Taqman probes and primers used for qPCR.
